# A *Pseudomonas fluorescens* type 6 secretion system is related to mucoidy, motility and bacterial competition

**DOI:** 10.1186/s12866-015-0405-9

**Published:** 2015-03-26

**Authors:** Victorien Decoin, Mathias Gallique, Corinne Barbey, Francois Le Mauff, Cecile Duclairoir Poc, Marc GJ Feuilloley, Nicole Orange, Annabelle Merieau

**Affiliations:** LMSM, Laboratoire de Microbiologie Signaux et Microenvironnement, Normandie Université, EA 4312, IRIB, Université de Rouen, IUT d’Evreux, 55 rue Saint Germain, 27000 Evreux, France; GlycoMEV, Laboratoire de Glycobiologie et Matrice Extracellulaire Végétale, Normandie Université, EA 4358, Université de Rouen, Faculté des sciences, Batiment 20 Gadeau de Kerville, IRIB 76820 Mont Saint-Aignan, France

**Keywords:** *Pseudomonas fluorescens*, Type 6 secretion system, Hcp protein, Competitive inhibition, Motility, Mucoidy, Exopolysaccharides

## Abstract

**Background:**

*Pseudomonas fluorescens* strain MFE01 secretes in abundance two Hcp proteins (haemolysin co-regulated proteins) Hcp1 and Hcp2, characteristic of a functional type 6 secretion system. Phenotypic studies have shown that MFE01 has antibacterial activity against a wide range of competitor bacteria, including rhizobacteria and clinically relevant bacteria. Mutagenesis of the *hcp2* gene abolishes or reduces, depending on the target strain, MFE01 antibacterial activity. Hcp1, encoded by *hcp1*, may also be involved in bacterial competition. We therefore assessed the contribution of Hcp1 to competition of *P. fluorescens* MFE01 with other bacteria, by studying MFE01 mutants in various competitive conditions.

**Results:**

Mutation of *hcp1* had pleiotropic effects on the MFE01 phenotype. It affected mucoidy of the strain and its motility and was associated with the loss of flagella, which were restored by introduction of plasmid expressing *hcp1*. The *hcp1* mutation had no effect on bacterial competition during incubation in solid medium. MFE01 was able to sequester another *P. fluorescens* strain, MFN1032, under swimming conditions. The *hcp2* mutant but not the *hcp1* mutant conserved this ability. In competition assays on swarming medium, MFE01 impaired MFN1032 swarming and displayed killing activity. The *hcp2* mutant, but not the *hcp1* mutant, was able to reduce MFN1032 swarming. The *hcp1* and *hcp2* mutations each abolished killing activity in these conditions.

**Conclusion:**

Our findings implicate type 6 secretion of Hcp1 in mucoidy and motility of MFE01. Our study is the first to establish a link between a type 6 secretion system and flagellin and mucoidy. Hcp1 also appears to contribute to limiting the motility of prey cells to facilitate killing mediated by Hcp2. Inhibition of motility associated with an Hcp protein has never been described. With this work, we illustrate the importance and versatility of type 6 secretion systems in bacterial adaptation and fitness.

## Background

Environmental bacteria are in perpetual war against several competitors, and thus require weapons to conquer new territory. The type 6 secretion system (T6SS) of Gram-negative bacteria is an effector translocation apparatus resembling an inverted bacteriophage puncturing device [1-3]. It is involved in a broad variety of functions, including antibacterial activity [4,5] and bacterial communication [6]. For example, *Proteus mirabilis* uses the killing activity of T6SS for self-recognition: this T6SS seems to be activated when opposing *P. mirabilis* swarms meet. The result of its action is a visible boundary called the Dienes line [7,8].

The T6SS machinery comprises at least 13 proteins, the core components, and sometimes, additional proteins [9,10]. Some of the core component proteins can affect systems other than T6SS. Hcp proteins, extracellular components of this secretion machinery, are released into the medium, and therefore may serve as markers of a functional T6SS apparatus [11]. Silverman and colleagues demonstrated that *P. aeruginosa* Hcp are not only structural proteins but also play a crucial role as chaperone and receptor for T6SS effectors. Various Hcp proteins transport their own effectors and this effector selection by Hcp seems to be specific [12].

The diversity of T6SS regulation reflects the vast array of its functions. The *P. aeruginosa* H1-T6SS gene cluster exhibits posttranscriptional regulation involving two sensor kinases, RetS and LadS [13-15]. High concentrations of synthetic c-di-GMP have negative effects on sensor kinase RetS, leading to *P. aeruginosa* H1-T6SS being turned on [16]. It is likely that a similar regulatory pathway controls the expression of *P. protegens* Pf-5 and *P. syringae* pv. syringae T6SS gene clusters [17,18]. However, T6SS regulation involves systems that regulate other genes suggesting regulatory cross-talk between T6SS and other virulence factors.

Environmental bacteria are often surrounded by an extracellular matrix, forming a protective capsule called the glycocalyx [19]. This extracellular matrix is generally composed of bacterial exopolysaccharides (EPS). EPS are involved in a variety of functions, including microcolony formation, protection against bacteriophages and mucoid phenotypes. The mucoid phenotype of *Pseudomonas* sp. is believed to be a global adaptive stress response to adverse environmental conditions [20,21]. It is characterized by overproduction of EPS alginate leading to shiny, raised and opaque colonies. The mucoid phenotype is a major factor contributing to *P. aeruginosa* infection in cystic fibrosis (CF) patients [22,23] but is unstable *in vitro* [24]. Some *P. aeruginosa* CF isolates acquire a mucoid phenotype through mutation of the anti-sigma factor MucA, a negative regulatory factor that sequesters AlgU, a positive regulator of alginate production [22,25]. A proteomic study by Rao and coworkers found that mucoid *P. aeruginosa* strains do not express T6SS genes [26]. Unlike *P. aeruginosa*, some *Vibrio cholerae* strains are mucoid and have an active T6SS [27]. Some environmental *P. fluorescens* strains produce alginate or neutral and amino sugars which give a mucoid phenotype [28,29]. The *P. fluorescens* mucoid phenotype, like that in *P. aeruginosa*, was reported to be unstable [29]. The mucoid phenotype can occur following mutation of the negative regulator of the alginate biosynthetic operon, muc [30]. We previously characterized the strain MFE01, a mucoid environmental *P. fluorescens* isolate. It constitutively secretes two characteristic T6SS proteins at 28°C: Hcp1 and Hcp2. It also exerts antibacterial activity during contact on a solid surface with competitive bacteria; this activity is associated with Hcp2 [31]. The aim of this work was to study the role of Hcp1 and the link between T6SS and the mucoid phenotype of MFE01.

## Results and discussion

### Some mucoid *P. fluorescens* strains secrete Hcp abundantly

**Figure 1:**
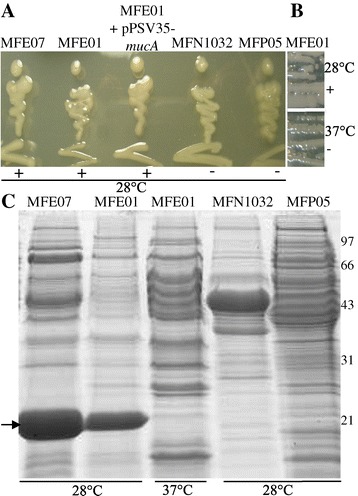
**Hcp secretion and mucoid phenotype in** ***P. fluorescens*** **strains. A**: Mucoidy was assessed on LB agar at 28°C. +: mucoid, −: non mucoid. **B**: Mucoidy at 28°C or 37°C of MFE01. +: mucoid, −: non mucoid. The images shown are representative of three assays. **C**: Concentrated supernatants of cultures in late exponential growth phase, grown at 28°C or 37°C, were analysed by SDS-PAGE (15% separation gel) and Coomassie staining. Bands (indicated by arrows) with an approximate molecular mass of 21 kDa were observed in MFE01 and MFE07 supernatants at 28°C. Mass spectrometry identified these major supernatant proteins as Hcp proteins.

The phenotypes of four *P. fluorescens* strains were observed after growth on LB agar plates at 28°C. Environmental strains MFE01 [31] and MFE07 (this study) had a stable shiny aspect, characteristic of mucoid phenotypes at 28°C. Skin strain MFP05 [32] and clinical strain MFN1032 [33] did not have a mucoid phenotype at this temperature (Figure 1A and B).

MucA is a negative regulator of the mucoid phenotype [22] in *Pseudomonas* species. We introduced the *mucA* gene of the *Pseudomonas protegens* strain Pf-5 (previously described as a *P. fluorescens* strain) [34] into MFE01. The expression of *mucA* did not switch-off mucoidy at 28°C, suggesting that the mucoid phenotype of MFE01 is not due to a mutation of the *mucA* gene or is MucA independent (Figure 1A). Scanlan and Buckling showed that the environmental *P. fluorescens* strain SBW25 has an unstable mucoid phenotype; although the genetic mechanisms of this phenotype remain unknown, they do not include mutations at many of the loci commonly reported to be involved in mucoid conversion, including *mucA* and *algU*. They also demonstrated that lytic phage exerts a selection pressure by selecting the mucoid phenotype [29].

We prepared supernatants of cultures of mucoid *P. fluorescens* strains MFE01 and MFE07 and found that these strains secreted large amounts of proteins identified as Hcp by Mass Spectroscopy (MS) (Figure 1C). The spot for the protein secreted by MFE01 matched significantly with gi: 398989489 (T6SS effector, Hcp1 family from *Pseudomonas* sp GM24) with a score of 120 for a score threshold of 84. The spot for the protein secreted by MFE07 spot matched significantly with gi:77458272 (hypothetical protein Pfl01_2045 from *Pseudomonas fluorescens* Pf0-1, Hcp1 family) with a score of 65 for a score threshold of 54. The non-mucoid *P. fluorescens* strains MFP05 and MFN1032 did not secrete detectable amounts of Hcp proteins into the extracellular medium.

The optimal growth temperature of MFE01 is 28°C but it can grow at 37°C. Clearly visible at 28°C, the mucoid phenotype and Hcp secretion were both switched off at 37°C, suggesting a common regulation of these two phenotypes (Figure 1B,C).

Unterweger and colleagues observed a correlation between the mucoid phenotype and Hcp secretion in *V. cholerae* smooth strains [27]. Sigma-54 controls T6SS genes transcription, so they introduced *vasH*, encoding a sigma-54 activator protein, into rough (non mucoid) *V. cholera* strains impaired in Hcp secretion: the expression of *vasH* restored the mucoid phenotype*,* but not the Hcp secretion. However, *vasH* can activate transcription of genes other than T6SS genes *via* sigma-54 [35,36] suggesting that the correlation between mucoidy and T6SS in *V. cholerae* may be due to a common regulation system.

### Hcp1 of *P. fluorescens* MFE01 is involved in mucoid phenotype

**Figure 2:**
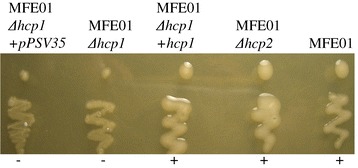
**Effects of** ***hcp1*** **and h** ***cp2*** **mutations on mucoid phenotype.** Representative images of mucoidy of MFE01, MFE01Δ*hcp1*, MFE01Δ*hcp2,* MFE01Δ*hcp1+pPSV35* and MFE01Δ*hcp1+hcp1* at 28°C on LB agar (n = 3). +: mucoid, −: non mucoid.

**Figure 3:**
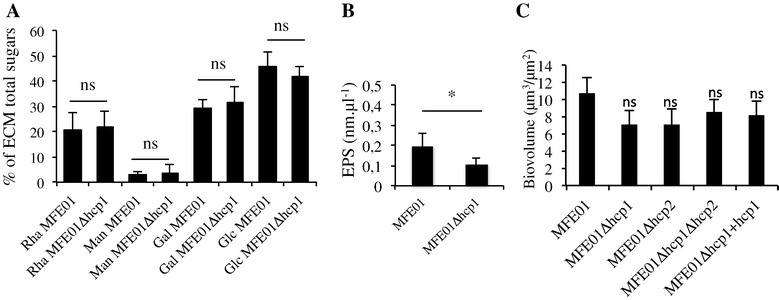
**Analysis of the sugar composition of the extracellular matrix and effect of** ***hcp1*** **and** ***hcp2*** **mutations on biofilm biovolume. A**: Relative quantity of major sugars (Rha = Rhamnose, Man = Mannose, Gal = Galactose and Glc = Glucose) determined by gas chromatography in extracellular matrix (ECM) of MFE01 and MFE01Δ*hcp1.* Values are mean results of four independent experiments, and the error bars represent standard error of the mean. Strains were compared pairwise by non-parametric Mann–Whitney two-tailed tests. ns: no significant difference. **B**: Gas chromatographic determinations of the absolute quantity of exopolysaccharides (EPS) in the extracellular matrix (ECM) of MFE01 and MFE01Δ*hcp1.* Values are mean results of four independent experiments and the error bars represent standard error of the mean. Strains were compared pairwise by non-parametric Mann–Whitney two-tailed tests. *p-value <0.05. **C**: Biovolume of biofilms. The biofilms were grown on glass surface at 28°C for 48 h under a flow of LB medium. Biovolumes were obtained by COMSTAT analyses. Values are mean results of at least five independent experiments and the error bars represent standard error of the mean. Statistical analyses were performed using Non-parametric Mann–Whitney Tests (two tailed). ns means no significant difference in biovolume (*p*-value >0.05) relative to the MFE01 biofilm biovolume assay.

We constructed *hcp* mutants, and found that the *hcp1* mutation (MFE01∆*hcp1* strain) lead to the loss of mucoidy at 28°C whereas the *hcp2* mutation (MFE01∆*hcp2* strain) did not affect the mucoid phenotype (Figure 2). The introduction of a plasmid carrying the *hcp1* gene into MFE01∆*hcp1* (MFE01∆*hcp1+hcp1* strain) restored the wild-type phenotype, suggesting a direct link between *hcp1* expression and mucoidy. We analysed and compared the extracellular matrices (ECM) of MFE01 and MFE01∆*hcp1*. The mucoid phenotype is a consequence of exopolysaccharide (EPS) accumulation [37], so we determined ECM sugar composition by Gas–liquid Chromatography (GLC). No significant difference between MFE01 and MFE01∆*hcp1* was observed concerning the proportion of characteristic sugars of the *Pseudomonas* EPS (rhamnose, mannose, galactose and glucose; Figure 3A). Thus, MFE01∆*hcp1* is able to secrete an EPS similar to that produced by MFE01. Nevertheless, the MFE01 ECM contained twice as much EPS as the MFE01∆*hcp1* ECM suggesting that the loss of the mucoid phenotype was a consequence of less EPS accumulation (Figure 3B). In *Pseudomonas aeruginosa*, biofilm formation and T6SS expression are negatively regulated by the sensor RetS and *ret*S mutants produce a hyperbiofilm phenotype [16]. Biofilm formation by MFE01 and mutants was then assayed. The moderate biofilm biovolume was not significantly different between MFE01 and MFE01∆*hcp1 or* MFE01∆*hcp2* mutants (Figure 3C). These findings indicate that mucoid phenotype and biofilm formation are not co-regulated by T6SS in MFE01.

**Figure 4:**
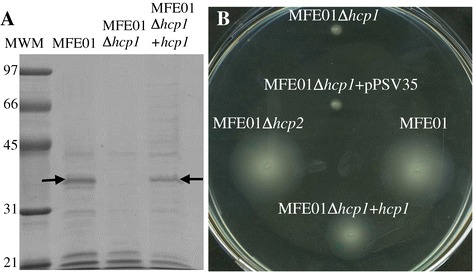
**Effect of** ***hcp1*** **and** ***hcp2*** **mutations on flagellin secretion into the extracellular matrix and on motility. A**: Protein fractions from the ECM of MFE01, MFE01*Δhcp1* and MFE01*Δhcp1+hcp1* were analysed by SDS-PAGE and Coomassie staining. Bands with approximate molecular mass ≈ 38 kDa, indicated by the arrows, were observed at the growth temperature of 28°C and were identified as flagellin proteins by MALDI-TOF (n = 3). Molecular weight markers are indicated. **B**: Swimming assay on LB in 0.3% agar at 28°C. MFE01, MFE01Δ*hcp2* and MFE01Δ*hcp1*+*hcp1* were motile but MFE01Δ*hcp1* and MFE01Δ*hcp1+* pPSV35 (empty plasmid used as control) were non motile (n = 3).

ECM protein fractions from supernatants of MFE01, MFE01∆*hcp1* and MFE01∆*hcp1+hcp1* were studied by SDS-PAGE (Figure 4A). Proteins of approximate molecular mass of 38 kDa were present in ECM extracts from MFE01 and MFE01∆*hcp1+hcp1* but not in MFE01∆*hcp1*. MS identified these 38 kDa proteins as flagellin proteins: they matched significantly with flagellin from *Pseudomonas moraviensis* (gi: 515142380) with a score of 94 for a score threshold of 86. Thus, the mucoid phenotype appears to involve the accumulation of both EPS and flagellin in ECM, which are perturbed by *hcp1* deletion. This was unexpected because the literature generally reports an inverse cross-talk between the mucoid phenotype and flagellar assembly. For example, the alternative sigma factor, σ22 (synonym AlgT or AlgU), is a positive regulator of alginate biosynthesis and a negative regulator of flagellum biosynthesis [38].

### *P. fluorescens* MFE01 Hcp1 is involved in motility

We examined motility of our various strains to elucidate the relation between flagellin accumulation in the ECM and flagellum functionality. MFE01, MFE01∆*hcp1,* MFE01∆*hcp2*, MFE01Δ*hcp1*+pPSV35 (empty vector control) and MFE01∆*hcp1+hcp1* were assayed for swimming; this confirmed that MFE01∆*hcp1* had lost motility that was restored by *hcp1* introduction *in trans*. The restoration of wild-type phenotype in MFE01∆*hcp1+hcp1* is inconsistent with a possible polar effect of the *hcp1* deletion (Figure 4B).

The type 3 secretion system is related to the flagellum [39], but little is known about the relation between T6SS and the flagellar regulon. The IcmF of *Vibrio cholerae*, a T6SS protein, is involved in motility [40]. IcmF is an inner-membrane protein of the T6SS found in numerous pathogens, and has been implicated in intracellular multiplication inside host cells [41,42]. In an avian pathogenic *E. coli*, *icmF* mutation impaired motility [43]; the authors indicate that this contrasts with other mutants in T6SS genes, notably *clpV* and *hcp*, which had no motility defects. Their findings suggest that the motility defect of the *icmF* mutant was not due to a general defect of motility because of a non-functional T6SS. IcmF was somehow involved in flagellar regulation. The restoration of the motility of MFE01∆*hcp1* by *hcp1* introduction *in trans* implies that the function of T6SS associated to *hcp1* expression is directly involved in motility in this strain.

### The *hcp1* mutant of *P. fluorescens* MFE01 retains virulence toward competitor bacteria

**Figure 5:**
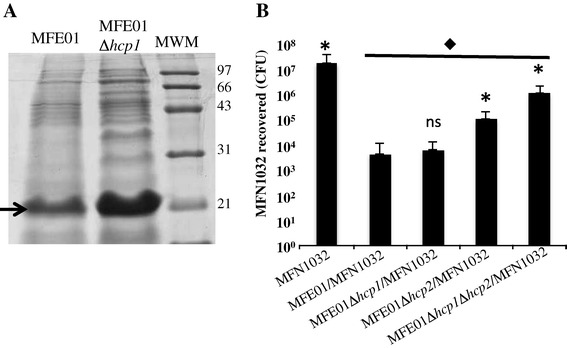
**Hcp secretion and killing activity of** ***P. fluorescens*** **MFE01 and derivatives strains. A**: Concentrated supernatants of MFE01 and MFE01Δ*hcp1* cultures were analysed by SDS-PAGE and Coomassie staining. Bands with a molecular mass similar to that of an Hcp protein (≈20 kDa), indicated by the arrow, were observed at a growth temperature of 28°C. MWM: molecular weight markers are indicated. **B**: Quantitative co-culture assays were performed. Prey cells (MFN1032 carrying pSMC21-*gfp*) were or were not mixed at ratio of 1:5 with *P. fluorescens* MFE01, MFE01Δ*hcp1*, MFE01Δ*hcp* *1*Δ*hcp2* and MFE01Δ*hcp2*; after 4 h at 28°C, MFN1032 cfu were counted (n = 4, the error bars represent standard error of the mean). * Indicates a significant difference in MFN1032 cfu (*p*-value <0.05) relative to the MFN1032/MFE01 assay; ns means no significant difference. ♦ indicates a significant difference in MFN1032 cfu (*p*-value <0.05) relative to the MFN1032 alone control assay.

The Hcp concentration was slightly lower in the supernatant of MFE01∆*hcp1* than that of the wild-type MFE01 at 28°C (Figure 5A). This is consistent with our previous observations [31]: most of the Hcp secreted by MFE01 is produced from the expression of the *hcp2* gene. We have also demonstrated that MFE01 has a killing activity against various Gram-negative bacteria on solid medium. MFE01Δ*hcp2* reduced prey cell populations, but significantly less than MFE01 indicating that Hcp2 contributes to the killing activity, and that another factor is also involved. To determine whether Hcp1 could be this other factor, we co-cultured mutant MFE01Δ*hcp1* and the prey MFN1032 on a filter on solid media for 4h at 28°C. There was no significant difference of the MFN1032 population between the co-culture with wild-type MFE01 and the co-culture with MFE01∆*hcp1* (Figure 5B). Thus, Hcp1 was not the key factor in this antibacterial activity even if it contributed to killing. Whereas, the MFE01∆*hcp2* mutant had significantly less antibacterial activity than MFE01 and MFE01∆*hcp1*. The double mutant, MFE01∆*hcp1*∆*hcp2*, was even less bactericidal than MFE01∆*hcp2* but nevertheless reduced the MFN1032 population. Another system independent of Hcp1 and Hcp2 seems to be involved in killing activity.

### *P. fluorescens* MFE01 inhibits the motility of *P. fluorescens* strain MFN1032

**Figure 6:**
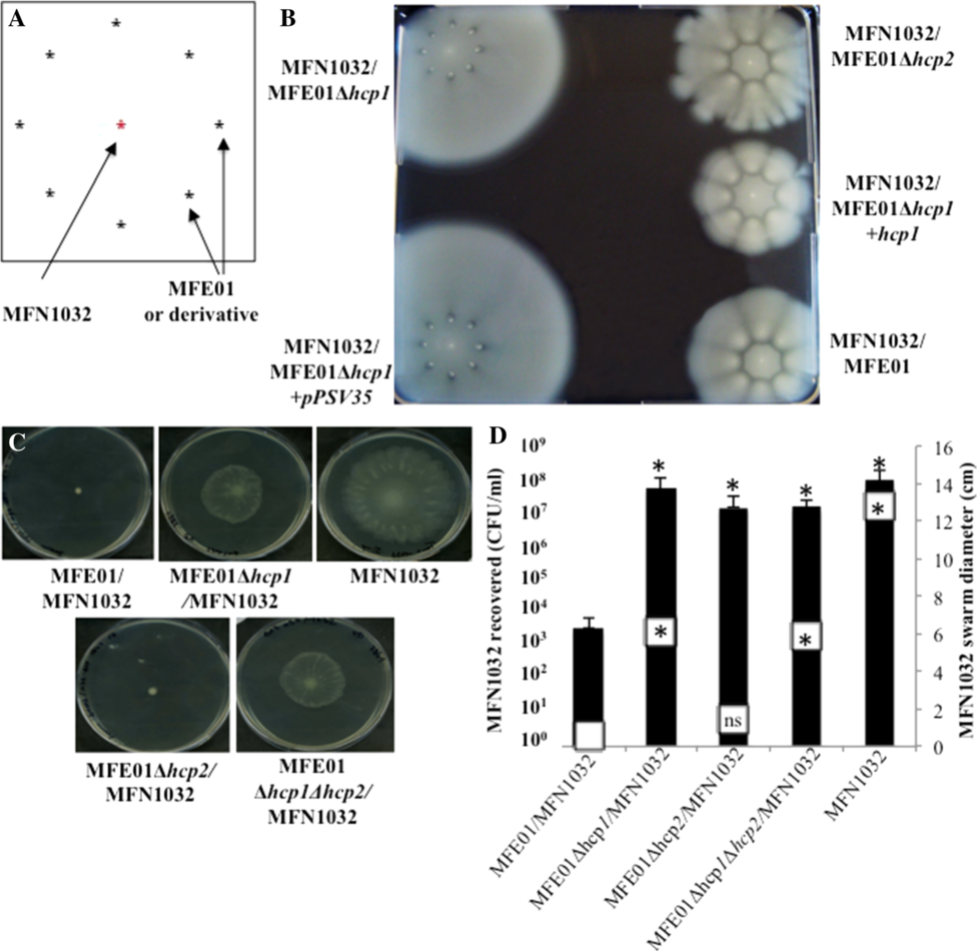
**Effects of** ***P. fluorescens*** **MFE01 and its mutants on competitive bacteria. A**: Schematic drawing of the assay: *P. fluorescens* MFN1032 was inoculated into the centre of a ring formed by MFE01 or a mutant on LB medium in 0.3% agar. On this medium MFN1032 and ME01 can swim. **B**: Swimming of MFN1032 in competition with MFE01 or mutants. Results after incubation at 28°C, 24 h. The images shown are representative of three assays. **C**: Co-cultures were performed on swarming medium (LB in 0.6% agar) at 28°C, 24h. On this medium MFN1032 can swarm but ME01 cannot. Prey cells (MFN1032) were or were not mixed with *P. fluorescens* MFE01 or its derivatives at ratio 1:5. The images shown are representative of three assays. **D**: Quantitative black histograms represent survival of MFN1032 and white squares the MFN1032 swarm diameter (n = 4, error bars represent standard error of the mean). * Indicates a significant difference of the MFN1032 cfu (*p*-value <0.05) relative to the MFN1032/MFE01 assay. ns: no significant difference.

The killing activity mediated by T6SS during swarming in *P. mirabilis* involves self-recognition (the Dienes effect), and we therefore studied the behaviour of MFE01 in conditions permitting its motility [7,8]. In swimming conditions (in 0.3% agar), *P. fluorescens* MFN1032 was spotted into the centre of a ring formed by MFE01 or its mutants (Figure 6A). MFE01, MFE01∆*hcp1+hcp1* and MFE01∆*hcp2* inhibited MFN1032 motility, but swimming MFN1032 spread through the MFE01∆*hcp1* and*,* MFE01∆*hcp1+pPSV35* mutants (Figure 6B). The *hcp1* mutation thus abolished MFE01 confining MFN1032*.* Introduction of the native *hcp1* gene in *trans* restored the inhibitory activity on MFN1032 swimming. Thus, in these swimming conditions, *hcp1* expression seems required for *P. fluorescens* MFE01 to control the swimming of *P. fluorescens* MFN1032. In these conditions, MFE01∆*hcp1* lacked motility whereas MFE01 was motile (Figure 4B). The confinement of MFN1032 could be simply due to the formation of a physical barrier by the motile MFE01 whereas non-motile MFE01∆*hcp1* could not form this barrier*.*

So we therefore conducted co-cultures overnight on 0.6% LB agar, conditions allowing the swarming of MFN1032. On these plates, MFE01 and mutants were unable to swarm because MFE01 lacks surfactants essential for *P. fluorescens* swarming [44]. MFE01 and MFE01∆*hcp2* clearly inhibited MFN1032 swarming, whereas MFE01∆*hcp1* and MFE01∆*hcp1*∆*hcp2* had much less effect on MFN1032 swarming (Figure 6C,D). This indicates that the inhibition of motility observed was not due to MFE01 or its derivatives forming a physical barrier. Also, diameter of the MFN1032 swarming was reduced, albeit to a lesser extent, by the double mutant MFE01∆*hcp1*∆*hcp2* providing further evidence for another unidentified inhibitory factor. These experiments are uninformative about whether the decrease in swarming diameter is due to a killing activity or an immobilization activity.

We consequently assayed the bactericidal activity of MFE01 and its mutants by counting MFN1032 cells in each swarming condition (Figure 6D). The MFN1032 population underwent 5-logs drops after co-culture with wild-type MFE01. Co-culture with MFE01∆*hcp1* and MFE01∆*hcp2* did not significantly decrease the MFN1032 population. We assumed that when co-cultured with MFE01∆*hcp1,* MFN1032 cells could swarm and thereby escape from the killing activity mediated by Hcp2, which requires close contact. When in co-culture with MFE01∆*hcp2,* MFN1032 could not be able to escape but the killing activity is abolished by *hcp2* mutation*.* In antibacterial activity assays on solid media (Figure 5B), the MFN1032 cells were immobilized on a filter, and therefore unable to escape the killing activity of the *hcp1* mutant.

These results suggest that MFE01 needs Hcp1 and Hcp2 to kill MFN1032 in conditions of motility: Hcp1 could reduce the motility of prey cells and thereby facilitate killing by Hcp2.

### Phenotypes assigned to Hcp1 and Hcp2 are associated to a T6SS

**Figure 7:**
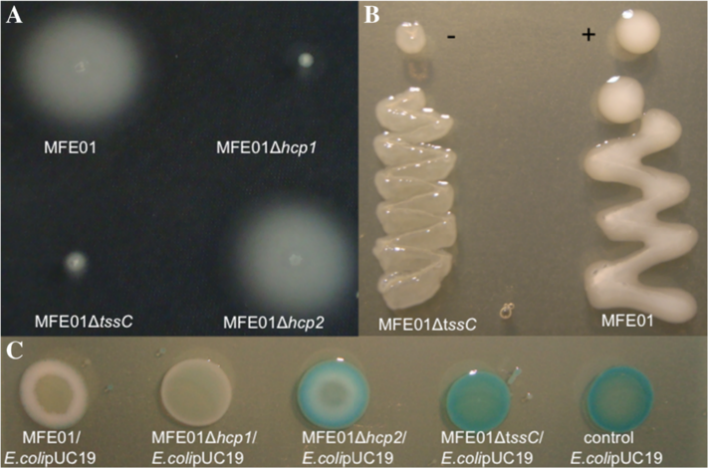
**Phenotypes of the MFE01**∆***tssC*** **mutant. A**: Swimming assay on LB in 0.3% agar at 28°C during 24 h. MFE01 and MFE01Δ*hcp2* were motile contrary to MFE01Δ*hcp1* and MFE01Δ*tssC* strains (n = 3). **B**: Representative images of mucoidy of MFE01 and MFE01Δ*tssC* on LB agar at 28°C, 24 h (n = 3). +: mucoid, −: non mucoid. **C**: Effects of *P. fluorescens* MFE01 and its mutants on competitive *E.coli* on solid medium. Co-cultures were performed on LB medium supplemented with X-Gal (40 μg/ml) at 28°C during 24 h. Prey cells (*E.coli DH5αmcr c*ontaining pUC19) were or were not mixed with *P. fluorescens* MFE01 or its derivatives at ratio 1:1. Blue is due to X-Gal degradation by *E.coli*pUC19*.* The images shown are representative of three assays.

Analysis of MFE01 draft genome is under way. Preliminary results indicated that this genome contains only one T6SS cluster exhibiting all core component genes but *hcp* genes. The two *hcp* genes, *hcp1* and *hcp2,* are separately located outside this cluster and are associated with *vgr*G genes. Into this unique T6SS we identified a gene coding for a putative protein corresponding to a protein member of EvpB/VC_A108 family (TIGR03355). This putative protein matched with the WP_016772499.1 sequence (MULTISPECIES: type VI secretion protein [*Pseudomonas*], with 99% identity and coverage of 100%). These proteins are described as T6SS needle sheath proteins TssC and are essential components of this system. MFE01∆*tssC,* a mutant disrupted in this T6SS core component gene, was non motile in swimming conditions and non-mucoid at 28°C as well as MFE01∆*hcp1* (Figure 7A and B) whereas MFE01∆*hcp2* conserved the wild type phenotype (mucoid and motile). During co-culture on LBG containing X-gal at 28°C with *E.coli* containing pUC19 (*E.coli*pUC19), MFE01∆*hcp2* and MFE01∆*tssC* allowed *E.coli*pUC19 growth (blue spots) whereas MFE01∆*hcp1* and MFE01 inhibited *E.coli*pUC19 growth (white spots) (Figure 7C). ME01∆*tssC* phenotype seems similar to a patchwork of MFE01∆*hcp1* and MFE01∆*hcp2* phenotypes. These results provide a strong confirmation of Hcp1 and Hcp2 involvement in a T6SS.

## Conclusion

In this work, we further illustrate the importance and versatility of the T6SS in bacterial adaptation and fitness. We demonstrated cooperation between two different Hcp proteins in bacterial competition. The observations indicate that T6SS associated with Hcp1 secretion has pleiotropic effects on *P. fluorescens* MFE01: it affects mucoidy, motility and competitor inhibition in motility conditions. The relationships between T6SS and flagellin and between T6SS and mucoidy have not yet been fully elucidated. Nevertheless, we describe some of the mechanisms associated with MFE01 motility and inhibition of prey cell motility. This suggests that some effectors, that are used to inhibit competitor motility, could be accumulated in MFE01∆*hcp1*, in absence of Hcp1, and consequently might inhibit the mutant’s own motility. Analysis of MFE01 draft genome is under way. Numerous mutants should be constructed to analyse function of unknown putative genes surrounding these *hcp* genes to ensure an informative genomic annotation and to explain how this T6SS could impact mucoidy and motility.

## Methods

### Bacterial strains, plasmids and culture conditions

All strains and plasmids used are listed in Table 1. All bacterial strains were grown in LB medium with shaking (180 rpm). *Pseudomonas fluorescens* strains were grown at 28°C or 37°C and *Escherichia coli* at 37°C. Media were supplemented with antibiotics as appropriate: kanamycin (Km) 50 μg/ml (*E. coli*) or 200 μg/ml (*P. fluorescens*); tetracycline (Tc) 15 μg/ml; gentamicin (Gt) 15 μg/ml (*E. coli*), or 50 μg/ml or 100 μg/ml (*P. fluorescens* in liquid and solid media, respectively).

**Table 1:** **Plasmids and strains used in the study**

**Strains or plasmids**	**Relevant characteristics**	**Reference/source**
*P. fluorescens*		
MFE01	Air isolate, Rif^R^	[31]
MFE01∆*hcp2*	MFE01 with early stop codon in *hcp2*	[31]
MFE01∆*hcp1*	MFE01 with *hcp1* disruption	This study
MFE01∆*hcp1 + hcp1*	MFE01∆*hcp1* with pPSV35 carrying the wild-type *hcp1* gene	This study
MFE01∆*hcp1*∆*hcp2*	MFE01 with *hcp1* and *hcp2* mutations	This study
MFE01∆*tssC*	MFE01 with *tssC* disruption	This study
MFN1032	Clinical strain	[33]
MFP05	Skin isolate	[32]
Pf-5	Plant isolate	[34]
MFE07	Air isolate	This study
*Escherichia coli*		
*DH5αmcr*	General cloning strain	LMSM collection
S17.1	RP4-2-Tc::Mu, *aph*::Tn7, recA, SmR, donor strain for conjugation	[48]
Vectors		
pPSV35	*P. aeruginosa oriV*, *lacIq mob+*, P*lac*UV5, pUC18 MCS, expression vector, Gm^R^	[49]
pSMC21*-gfp*	Replicative plasmid, Km*R*, *gfp*	[46]
pME3087	Suicide plasmid, Tc^R^	[47]
pUC19	Replicative plasmid, *lacZ*, Ap^R^	Invitrogen®

### Hcp secretion analysis

Hcp secretion was studied by harvesting the supernatants by centrifuging the cultures at 5000 × *g* for 10 minutes at 20°C and passing them through a Millipore membrane with 0.22 μm pores. TCA (trichloroacetic acid, Sigma-Aldrich) was added to the supernatant to a final concentration of 10% and the mixture was incubated overnight at 4°C. The supernatant was removed by centrifugation at 13000 × *g*, for 30 minutes at 4°C. The protein pellet was washed twice with 5 ml of 20 mM Tris base (VWR) in cold acetone (Merck) and centrifuged at 13000 × *g*, for 30 minutes at 4°C. The dry pellet was then resuspended in distilled water. Proteins were separated by sodium dodecyl sulphate polyacrylamide gel electrophoresis (SDS-PAGE). Briefly, samples were mixed with an equal volume of 2 × Laemmli sample buffer (with β-mercaptoethanol), boiled for 5 min at 100°C and then cooled to room temperature before loading.

### Mucoid phenotype and swimming assays

Strains were plated on LB agar with 0.1 mM IPTG and incubated for 24 h at 28°C; the colonies were then examined for the mucoid phenotype. Swimming assays were performed as previously described [45].

### Swarming killing assay

The protocol described by Alteri and colleagues [8] was used with minor modifications. Aliquots of 5 μl of a mixture of strains at a ratio of 5:1, MFE01 or mutants: MFN1032 containing plasmid pSMC21*-gfp* [46] were spotted onto LB 0.6% agar with 0.1 mM IPTG. The plates were incubated overnight at 28°C, and the entire swarm was collected, and serially diluted. Aliquots of 10 μl of each dilution were plated on LB agar supplemented with 200 μg kanamycin and colony forming units counted.

### Motility inhibition

Overnight cultures were centrifuged at 3000 × *g*, for 5 minutes at room temperature. The cell pellets were collected on toothpicks which was used to stab 0.3% agar LB plates, with 0.1 mM IPTG; the plates were then incubated at 28°C overnight.

### Antibacterial competition assay

Antibacterial competition assays in solid media were performed as described by Decoin and coworkers [31]. To ensure pSMC21*-gfp* stability (presence or absence) in our conditions, MFN1032 containing pSMC21*-gfp* was cultivated in LB medium containing kanamycin until OD_580 nm_ of 1. Serial dilutions were then plated on LB plates without Kanamycin during 24 h at 28°C. Colonies were scrapped and suspended in NaCl 9 g/L and adjusted to OD5_580 nm_ of 1. Serial dilutions were plated on selective (with kanamycin) and non-selective (without kanamycin) plates and counting. Statistics were done by pairwise strain comparisons (non-parametric Mann–Whitney-two tailed Test): no significant difference was observed between the two conditions (p-value >0.05, n = 6).

For *E.coli* growth inhibition, *Pseudomonas fluorescens* MFE01 and mutants were cultivated at 28°C in LB medium with shaking at 180 rpm overnight. *Escherichia coli DH5αmcr* transformed with pUC19 (*E.coli*pUC19), allowing blue detection on medium containing X-Gal, was cultivated at 37°C in LB with ampicillin (50 μg/ml) with shaking at 180 rpm overnight. The OD_580 nm_ was adjusted to 0.5 and the strains were mixed at a 1:1 ratio. A volume of 20 μl was spotted in LB agar plates supplemented with X-Gal (40 μg/ml) and incubated overnight at 28°C.

### Disruption of the *hcp1* gene *in P. fluorescens* MFE01

MFE01∆*hcp1* was generated by deletions in the 5′ (44 bp) and 3′ extremities (18 bp) of *hcp1*. 5′ and 3′ truncated *hcp1* was generated by PCR with the Hcp3F and Hcp4R primers (see Table 2). The resulting truncated *hcp1* construct was introduced between the *Eco*RI and *Hind*III sites (blunt-ended) of the transferable suicide plasmid pME3087 (6.9 Kb) [47]. The resulting plasmid, pME3087∆*hcp1,* was verified by sequencing and was then transferred into MFE01 by biparental mating: *E. coli* S17-1 [48] containing pME3087∆*hcp1* and recipient MFE01 cells were mixed and spotted onto LB agar plates and incubated overnight at 37°C. The mating mixture was then suspended in 1 ml of sterile NaCl 9 g/L and 0.1 ml aliquots were spread on LB agar plates supplemented with tetracycline (12 μg/ml), to select for the presence of the integrated plasmid, and rifampicin (25 μg/ml), to kill the *E. coli* S17-1 donor bacteria and to ensure the selection of MFE01. The resulting *hcp1* mutant (containing two truncated *hcp1* genes) was named MFE01∆*hcp1.*

**Table 2:** **Oligonucleotides used for this study**

**Primer name**	**Primer sequence (5′-3′)**
PFL_1449mucAF	ATAATAAGAGCTCATGAGTCGTGAAGCCCTGC
PFL_1449mucAR	ATAATAATCTAGATTAGCGGTTTTCCAGGCTTG
Hcp1F	ATGGCAACACCAGCGTACATG
Hcp2R	TTAAACGACTGGAGCACGCCA
Hcp3F	CCTGATCACTGCCGGCGCTT
Hcp4R	ACGCCAGTCATCGGAACCCG
muta1tssC	CTGAGACTCCAGTAGCCAAG
muta2tssC	AAGCTTTCCAGACCGAAGAAATACTTCGTGGGTCCAGGTGAT
muta3tssC	ATCACCTGGACCCACGAAGTATGAAGGCTTCATCTCCCTGAC
muta4tssC	ATGTCATTGAGATCGGGCAA

### Disruption of the *hcp2* gene in *P. fluorescens* MFE01∆*hcp1* and disruption of the *tssC* gene in *P. fluorescens* MFE01

A markerless *hcp2* mutation was introduced into MFE01∆*hcp1* strain by the protocol described by Decoin and co-workers [31]. The resulting strain was named MFE01∆*hcp1*∆*hcp2.* The same protocol with PCR modifications was used to introduce a markerless *tssC* mutation into MFE01 strain. This *tssC* deletion was achieved by PCR with the muta1tssC and muta2tssC primers (<650 bp product) or the muta3tssC and muta4tssC primers (<650 bp product) (Table 2). The PCR products obtained corresponded to the upstream and downstream parts, respectively, of the *tssC* gene of MFE01, each carrying an overlapping sequence at the end. PCR parameters were as follows: annealing temperature, 62°C, extension time, 45 s; 35 cycles. A third PCR was then carried out in which the overlapping sequences of the two first products were hybridized together allowing the *tssC* deletion after PCR with the muta1tssC and muta4tssC primers. The PCR parameters were as follows: annealing temperature, 62°C; extension time, 1 m 45 s and 35 cycles. The mutant containing the *tssC* deletion was verified by DNA sequencing and named MFE01∆*tssC.*

### Heterologous expression of *mucA* in MFE01

The *mucA* (PFL_1449) sequence was amplified with the PFL_1449mucAF and PFL_1449mucAR primers (Table 2) by standard PCR from genomic DNA from *P. protegens* Pf-5. The PCR conditions were as follows: an annealing temperature of 64°C, an extension time of 30 s and 25 cycles. The polymerase used was Phusion® High-Fidelity DNA polymerase (NEB). The PCR product was digested with *Sac*I (NEB) and *Xba*I (NEB) and ligated into pPSV35 [49] digested with the same enzymes. *E. coli* was transformed with the ligation product and the resulting plasmid was checked by PCR. Fresh MFE01 colonies were washed twice in cold sterile distilled water and transformed with 5 μl of plasmid DNA by electroporation in 1 mm cuvette at 1.8 kV (Savant electroporater). LB was then added and the mix incubated for 1 h at 28°C with shaking (180 rpm). The samples were then plated on LB agar supplemented with gentamicin and with 0.1 mM IPTG.

### Insertion of the *hcp1* gene into pPSV35 and construction of strain MFE01∆*hcp1*+*hcp1*

The *hcp1* gene was amplified from the *P. fluorescens* MFE01 genome with the Hcp1F and Hcp2R primers (Table 2). The PCR conditions were as follows: an annealing temperature of 59°C, an extension time of 30 s and 25 cycles. The polymerase used was the Phusion® High-Fidelity DNA polymerase (NEB). The amplified fragments were inserted into the pPSV35 shuttle vector at *Sma*I site by blunt-end ligation, and the resulting pPSV35-*hcp1* was used to transform *E. coli DH5αmcr* cells by electroporation. Plasmid DNA was isolated using the QIAprep Spin Miniprep Kit (Qiagen) and checked by PCR and asymmetric digestion with *BamHI* (NEB) to verify the orientation of the insert. MFE01∆*hcp1* was transformed with pPSV35-*hcp1* by electroporation as described in the section “Heterologous expression of *mucA* in MFE01”. The resulting strain was called MFE01∆*hcp1*+*hcp1*.

### Preparation and purification of extracellular matrix (ECM) containing exopolysaccharides (EPS)

Strains were tested for EPS production on LB agar at 28°C. ECM was extracted as described by Dignac and colleagues with modifications [50]. Biomass was harvested and suspended in NaCl 9 g/L then sonicated at 37 Watts for 30 sec on ice and centrifuged (20,000 × *g* for 30 min). Clear supernatants were collected and three volumes of ice-cold ethanol were added; the samples were incubated for 24 h at 4°C, and EPS were collected by centrifugation (7000 × *g*, 30 min). The EPS-containing pellets were resuspended in water and lyophilised.

### Analysis of the monosaccharide composition of EPS by gas chromatography – flame ionization

Lyophilised EPS were hydrolysed by treatment with 2M trifluoroacetic acid for 2 hours at 110°C. Monosaccharides were then derivatised: methanol-1M HCl (Supelco) was added and the samples incubated at 80°C overnight; then a mix of hexamethyldisiloxan:trimethyldisiloxan:pyridine (3:1:9, Supelco) was added and the samples incubated at 110°C for 20 minutes. The resulting derivatives were dried then dissolved in 1 ml of cyclohexane and injected into the 3800 GC system equipped with a CP-Sil5-CB column (Agilent Technologies). Elution was performed with the following gradient of temperature: 120°C to 160°C at a rate of 10°C per minute, 160°C to 220°C at a rate of 1.5°C per minute, 220°C to 280°C at a rate of 20°C per minute. Quantification was based on the internal standard and response factor determined previously for each monosaccharide.

### Biofilm formation

Pure culture biofilms were grown in continuous-culture three-channel flow cells (channel dimensions, 1 by 4 by 40 mm). The system was assembled and prepared as follows: system sterilization during 4 h with bleach at 1.2% and rinsing with sterile NaCl 9 g/L. Before flow chamber inoculation, LB medium was injected into the system at 28°C. Overnight cultures were centrifuged at 8 000 × g during 5 min at room temperature. Pellets were recovered and washed twice with 2 mL of sterilized physiological water. Channels were inoculated with 1 mL of pure bacterial suspension at DO_580_nm = 0.1. Bacteria were allowed to attach to the glass surface (microscope coverslip, VWR International, Fontenay sous Bois, France) during 2 h at 28°C under static conditions. Biofilm growth was then performed under a constant flow of LB medium with antibiotics at appropriate concentration if necessary (Wakson Marlow 205S, 2.5 mL/h) for 48 h at 28°C.

### Confocal laser scanning microscopy (CLSM) and image analyses

Microscopic observations were performed with a LSM 710 system, Zeiss, Germany by using a 63x oil immersion objective. Biofilms were observed after a 15 min incubation with 5 μM Syto 9® green fluorescent nucleic acid stain (life technologies) (excitation and emission λ, 488 nm and 510 nm, respectively). Biofilm stacks were analysed with COMSTAT software. The calculated parameter was the biovolume, which is the volume of bacteria (in μm^3^) per μm^2^ of glass surface. The results were the mean of at least five independent experiments.

### Mass spectrometry analysis

Mass spectroscopy (MS) analyses were performed with a MALDI-TOF AutoflexIII (Brucker) in positive ion mode as described by Barbey *et al.* [51]. Statistical analyses of the sequences involved determining the probability based on Mowse score with MASCOT software (peptide tolerance = 100 ppm and mass values = MH+). A *p*-value of less than 0.05 was considered significant. The criteria used to accept a protein identification based on peptide mass fingerprinting (PMF) data included a score probability greater than a score threshold defined by MASCOT software.

### Statistical analysis

Non-parametric Mann–Whitney Tests (two tailed) with GraphPad Prism version 6.0 (La Jolla, CA) were used for statistical analyses. A *p*-value <0.05 was considered to be statistically significant.
